# Ability of online drug databases to assist in clinical decision-making with infectious disease therapies

**DOI:** 10.1186/1471-2334-8-153

**Published:** 2008-11-06

**Authors:** Hyla H Polen, Antonia Zapantis, Kevin A Clauson, Jennifer Jebrock, Mark Paris

**Affiliations:** 1 Nova Southeastern University, College of Pharmacy, Palm Beach Gardens, USA; 2 Pharmacy Practice Department, Nova Southeastern University, College of Pharmacy, Fort Lauderdale, USA; 3 Pharmacy Practice Department, Nova Southeastern University, College of Pharmacy – West Palm Beach, Palm Beach Gardens, USA; 4 Broward General Medical Center and Nova Southeastern University, College of Pharmacy, Fort Lauderdale, USA; 5 Infectious Disease Clinics, Palm Beach County Health Department, Delray Beach, USA

## Abstract

**Background:**

Infectious disease (ID) is a dynamic field with new guidelines being adopted at a rapid rate. Clinical decision support tools (CDSTs) have proven beneficial in selecting treatment options to improve outcomes. However, there is a dearth of information on the abilities of CDSTs, such as drug information databases. This study evaluated online drug information databases when answering infectious disease-specific queries.

**Methods:**

Eight subscription drug information databases: American Hospital Formulary Service Drug Information (AHFS), Clinical Pharmacology (CP), Epocrates Online Premium (EOP), Facts & Comparisons 4.0 Online (FC), Lexi-Comp (LC), Lexi-Comp with AHFS (LC-AHFS), Micromedex (MM), and PEPID PDC (PPDC) and six freely accessible: DailyMed (DM), DIOne (DIO), Epocrates Online Free (EOF), Internet Drug Index (IDI), Johns Hopkins ABX Guide (JHAG), and Medscape Drug Reference (MDR) were evaluated for their scope (presence of an answer) and completeness (on a 3-point scale) in answering 147 infectious disease-specific questions. Questions were divided among five classifications: antibacterial, antiviral, antifungal, antiparasitic, and vaccination/immunization. Classifications were further divided into categories (e.g., dosage, administration, emerging resistance, synergy, and spectrum of activity). Databases were ranked based on scope and completeness scores. ANOVA and Chi-square were used to determine differences between individual databases and between subscription and free databases.

**Results:**

Scope scores revealed three discrete tiers of database performance: Tier 1 (82-77%), Tier 2 (73-65%) and Tier 3 (56-41%) which were significantly different from each other (p < 0.05). The top tier performers: MM (82%), MDR (81%), LC-AHFS (81%), AHFS (78%), and CP (77%) answered significantly more questions compared to other databases (p < 0.05). Top databases for completeness were: MM (97%), DM (96%), IDI (95%), and MDR (95%). Subscription databases performed better than free databases in all categories (p = 0.03). Databases suffered from 37 erroneous answers for an overall error rate of 1.8%.

**Conclusion:**

Drug information databases used in ID practice as CDSTs can be valuable resources. MM, MDR, LC-AHFS, AHFS, and CP were shown to be superior in their scope and completeness of information, and MM, AHFS, and MDR provided no erroneous answers. There is room for improvement in all evaluated databases.

## Background

Timely access to clinical decision support tools (CDSTs) has proven beneficial in selecting appropriate treatment options that result in improved therapeutic outcomes [1-6]. The use of such aids as personal digital assistants (PDAs), computerized physician order entry (CPOE), electronic health records (EHRs), and electronic databases has shown positive influence on patient morbidity and mortality, cost management and formulary compliance, and prevention of medication errors and related fatalities [3,7-13]. The arena of infectious disease (ID) is a complex and dynamic field with new treatment guidelines being adopted and innovative pharmaceutical options being introduced at a rapid rate. As such, ID management has a great potential for medication errors [14-16]. In fact, ID has benefited greatly from these innovative tools [3,17,18] both as a method to keep abreast of these rapid changes and as a mechanism to assist practitioners in understanding and embracing the most contemporary and appropriate therapies.

There is a dearth of information in the literature providing guidance to ID healthcare providers on the abilities of CDSTs, such as drug information databases, to provide the information most needed in this specialized practice setting. To date, no evaluations have been published that evaluated the ID content in online drug information databases, and only one study has been conducted that examined ID-specific drug information content in selected PDA programs. Miller et al. [19] evaluated four ID programs for PDAs, including: Epocrates ID, Johns Hopkins ABX Guide, Sanford's Guide to Antimicrobial Therapy, and Infectious Diseases and Antimicrobials Notes. This study focused on the salient features, advantages, disadvantages, and hardware and software requirements of each of these four databases. The evaluation of the scope and accuracy of the references' drug information was limited to a comparison of the monographs contained in each database to the information contained in the package insert for a single drug, *fluconazole*. Based on this narrow evaluation methodology, the study found that while each program contained the information physicians needed most at the point-of-care, such as dosing, adverse events, and drug interactions for antimicrobials, all of the applications had limited pharmacological and pharmacokinetic information. Some of the databases omitted important topics like pediatric dosing, contraindications, precautions, and adverse reactions. The authors concluded the use of PDA applications may decrease prescriptions errors, lead to significant improvements in patient outcomes, and cost reduction despite the identified shortcomings.

ID-specific PDA programs provide an important avenue for clinical decision support, especially for counsel needed at point-of-care. The question is: do they provide enough information about all aspects of pharmaceutical prescribing and management that specialty practitioners require? Utilization of online subscription or free drug information databases is an alternative to assist healthcare providers in making timely and accurate ID treatment determinations that encompass a plethora of medication topics relevant to patient care and treatment outcomes. ID practitioners have several products from which to choose and a multitude of factors must be weighed before making a decision on which online database best meets the user's needs. While several previous studies have been conducted to determine the abilities of online databases to satisfy the general drug information needs of healthcare providers [20-23], this study helps to elucidate the differences in selected online drug databases and compares the effectiveness of each CDST's ability to perform for an ID specialty setting. This study specifically aimed to evaluate the ability of online drug information databases to provide clinical decision support when answering infectious disease-specific queries.

## Methods

### Database selection

To be considered for inclusion, databases had to be accessible online and could either be designed as general drug information databases or ID-specific drug information databases. A list of references for consideration was compiled based on previous database studies [20-23], input from an expert panel, and utilization by current practitioners. Programs were included if the monographs were able to answer a diverse set of medication-related inquiries, including such topics as indications, adverse drug events, and drug interactions. Databases were excluded if they were intended exclusively for a particular practice setting other than ID, such as nursing, oncology, or pediatrics. Other references that were not designed primarily as drug information databases (e.g., Sanford's Guide to Antimicrobial Therapy and The 5 Minute Infectious Diseases Consult) were similarly excluded. Databases limited to answering a specific drug information question-type (e.g., drug interactions, compatibility/stability) were also omitted. Fourteen databases met the inclusion requirements: eight subscription and six freely accessible. Only one ID-specific database, Johns Hopkins ABX Guide, was identified that met the inclusion criteria. The subscription databases included American Hospital Formulary Service Drug Information (AHFS), Clinical Pharmacology (CP), Epocrates Online Premium (EOP), Facts & Comparisons 4.0 Online (FC), Lexi-Comp (LC), Lexi-Comp with AHFS (LC-AHFS), Micromedex (MM), and PEPID PDC (PPDC). The six freely accessible databases included: DailyMed (DM), DIOne (DIO), Epocrates Online Free (EOF), Internet Drug Index–RxList.com (IDI), Johns Hopkins ABX Guide (JHAG), and Medscape Drug Reference (MDR). Table 1 provides publisher and website details for each database included in this study.

**Table 1:** Database publishers and website addresses

**Database name**	**Abbreviation used in study**	**Publisher**	**Website**
American Hospital Formulary Service Drug Information	AHFS	American Society of Health-System Pharmacists	

Clinical Pharmacology	CP	Gold Standard	

DailyMed	DM	National Library of Medicine	

DIOne	DIO	Pharmacy OneSource, Inc.	

Epocrates Online Free	EOF	Epocrates, Inc.	

Epocrates Online Premium	EOP	Epocrates, Inc.	

Facts & comparisons 4.0 Online	FC	Wolters Kluwer Health	

Internet Drug Index	IDI	WebMD	

Johns Hopkins ABX Guide	JHAG	Johns Hopkins University	

Lexi-Comp	LC	Lexi-Comp, Inc.	

Lexi-Comp with American Hospital Formulary Service	LC-AHFS	Lexi-Comp, Inc.	

Medscape Drug Reference	MDR	Medscape, LLC	

Micromedex	MM	Thomson Healthcare	

PEPID PDC	PPDC	PEPID, LLC	

### Related databases

Lexi-Comp, Inc. offers two different versions of their drug information database: LC, which is a compilation of their standard drug monographs and LC-AHFS, which contains their standard monographs plus the information available in AHFS. Because of the potential similarity between AHFS, LC, and LC-AHFS, a subgroup analysis was performed to assess statistical differences between the databases.

### Category design

Five general treatment classifications were created based on a review of ID-related Anatomical Therapeutic Chemical (ATC) classification codes as listed on the 14^th ^World Health Organization (WHO) Model List of Essential Medicines: antibacterial, antiviral, antifungal, antiparasitic, and vaccination/immunization. These classifications were weighted based on importance of such factors as prevalence and incidence of infection type, morbidity and mortality data, and resistance patterns in the USA as reported by the Morbidity and Mortality Weekly Report (MMWR) and WHO. Within each of these classifications, 16 different drug information categories were designed, including dosage, interactions, emerging resistance, and spectrum of activity. Additionally, these categories were weighted based on their impact on direct patient care and importance to patient safety. For example, dosage and administration were considered more clinically relevant and thus weighted heavier, than categories less influential on direct patient care and safety such as cost and pharmacokinetics. Classification and category information, including weighting of each, is shown in Table 2.

**Table 2:** Weighting of classifications and categories

**CATEGORY**	**N (%)**	**VIRAL**	**BACTERIAL**	**FUNGAL**	**PARASITIC**	**VACCINATION/** **IMMUNIZATION**
**Dosing**	15 (10)	4	4	3	2	2
**Indication**	15 (10)	4	4	3	2	2
**Adverse reaction/event**	13 (9)	3	3	3	2	2
**Contraindications**	13 (9)	3	3	3	2	2
**Drug-Drug Interactions**	12 (8)	3	3	3	2	1
**Emerging Resistance**	12 (8)	4	4	2	1	1
**Administration**	10 (7)	3	3	2	1	1
**Mechanism of action**	9 (6)	2	2	2	2	1
**Food-Drug Interactions**	7 (5)	2	2	1	1	1
**Compatibility/Stability**	7 (5)	2	2	1	1	1
**Spectrum of Activity**	7 (5)	2	2	1	1	1
**Pregnancy/Lactation**	7 (5)	2	2	1	1	1
**Synergy**	6 (4)	2	2	1	1	0
**Drug-Herb Interactions**	5 (3)	1	1	1	1	1
**Cost**	5 (3)	1	1	1	1	1
**Pharmacokinetics**	4 (3)	1	1	1	1	0

**TOTAL**	**147 (100)**	**39**	**39**	**29**	**22**	**18**

### Question development

In order to have a greater likelihood of determining differences among the databases, a robust number of questions was needed. The most highly weighted categories were therefore assigned a total of 15 questions, and subsequent categories were populated with a stepwise reduction in the number of questions based on their weighted percentages. A set of 147 ID-specific question and answer pairs were developed and divided across the five ID classification categories and the 16 drug information categories. Answers were determined using manufacturer package inserts and primary literature, as well as gold standard references including the MMWR [24], Centers for Disease Control and Prevention (CDC) [25], Principles and Practices of Infectious Disease [26], and Natural Medicine Comprehensive Database [27]. The author-developed study design and question and answer set were reviewed by an external panel of ID physicians and pharmacists for accuracy and relevance to clinical practice. The question list was then finalized based on the panel recommendations. A sample of questions and answers is provided in Table 3.

**Table 3:** Sample questions and answers used in evaluation

How is tenofovir dosing adjusted for a hemodialysis patient? Tenofovir should be dosed at 300 mg every 7 days or after a total of approximately 12 hours of hemodialysis. Dose should be given after hemodialysis session.
Should sulfadiazine be used alone for toxoplasmosis prophylaxis? No. It should be used in conjunction with pyrimethamine and leucovorin.

What are the electrolyte abnormalities associated with Amphotericin B? Decreased magnesium, decreased calcium, and decreased or increased potassium.

Can nitazoxanide suspension be given safely to diabetic patients? Diabetics should be aware that the suspension contains 1.48 grams of sucrose per 5 mL.

What three drugs have cases reporting increased levels when administered with Fluzone? Phenytoin, warfarin, and theophylline.

What specific resistance profile has been documented in an HIV/HBV patient taking entecavir? M184V resistance substitution has occurred in the HIV strain.

What is used to reconstitute ertapenem for intramuscular administration? 3.2 mL of 1% Lidocaine HCl injection (without epinephrine).

What preservative does the Pneumovax pneumococcal vaccine contain? Phenol 0.25%.

How does spinal fluid concentrations of amikacin compare to serum concentrations in infants? Spinal fluid levels in normal infants are approximately 10 to 20% of the serum concentrations and may reach 50% when the meninges are inflamed.

What is the spectrum of activity of acyclovir? Herpes simplex virus types 1 (HSV-1), 2 (HSV-2), and varicella-zoster virus (VZV).

Can metronidazole be given to pregnant patients with trichomoniasis? It is contraindicated during the 1^st ^trimester of pregnancy due to lack of clinical evidence.

Against what viruses have foscarnet and ganciclovir shown a synergistic effect both in vitro and in vivo? Cytomegalovirus and herpes simplex virus type 2.

Why should willow bark be avoided 6 weeks after receiving Varivax? Because Reye's Syndrome has been reported following natural varicella infection.

### Database assessment

Databases were evaluated for their ability to answer each of the 147 questions and the completeness of the answers that the databases were able to provide. The presence or absence of the answer (scope) was determined, and a score of one was assigned for scope if the database provided the answer or a score of zero was assigned if the answer was absent. Answer completeness was determined using a 3-point scale, with three being the most complete and one being the least. Questions were structured in such a way that differences in completeness could be detected, often containing more than one part to the answer. Answers with only one part (e.g., Can valacyclovir be given to treat herpes encephalitis? No) would receive a three for completeness if the answer was present. If an answer had two components (e.g., What are the concerns with ceftriaxone administration in neonates? May displace bilirubin and cannot be administered with calcium-containing solutions due to risk of ceftriaxone-calcium precipitation) then completeness would be scored either a two if one answer was present or a three if both answers were present. For questions requiring three or more components to provide a complete answer, the completeness score was assigned a three if all components were present (e.g., What are the visual disturbances associated with voriconazole? Abnormal vision, color vision change, and/or photophobia). Completeness scores were only assigned if there was a score for scope. Assessments were made independently by at least two authors for two consecutive months ending in November 2007. In the three instances where the results were disparate, a consensus was reached on score assignation by the authors. Erroneous answers that were provided by the databases were also documented.

### Statistical analysis

Data were summarized using descriptive statistics to obtain rank order of databases based on scope and completeness scores. Inferential statistics were used to determine differences between individual databases and between subscription and free databases, via both ANOVA and Chi-square tests as appropriate. Tukey-Kramer's multiple comparison post-hoc tests were used to differentiate among databases. Similar analyses were conducted to determine statistical differences between AHFS, LC and LC-AHFS, as well as subscription and free versions of Epocrates. P values below 0.05 were considered statistically significant. This study was approved by the Health Professions Division Research Committee of Nova Southeastern University.

## Results

### Scope

Pair-wise comparisons of scope scores revealed three discrete tiers of database performance including: Tier 1 (Scope 82-77%), Tier 2 (Scope 73-65%) and Tier 3 (Scope 56-41%) which were all significantly different from each other (p < 0.05). The top tier performers: MM (82%), MDR (81%), LC-AHFS (81%), AHFS (78%), and CP (77%) answered significantly more questions when compared to the other databases (p < 0.05). The middle group of database scores (Tier 2) was: FC (73%), IDI (71%), DIO (65%), DM (65%), and LC (65%), and the lowest tier databases were: JHAG (56%), EOP (47%), EOF (46%), and PPDC (41%). Full details for database scores for scope across all categories and databases are included in Table 4.

**Table 4:** Scope of databases

**CATEGORY**	**N **(147)	**AHFS**	**CP**	**DIO**	**DM**	**EOF**	**EOP**	**FC**	**IDI**	**JHAG**	**LC**	**LC-AHFS**	**MDR**	**MM**	**PPDC**
Dosing	15	12	11	12	10	6	6	12	12	8	12	12	12	13	6
Indication	15	13	11	11	5	6	6	9	7	11	13	13	14	15	7
Adverse Reaction/Event	13	9	12	8	10	6	6	11	11	8	8	9	9	11	4
Contraindications	13	12	9	10	8	8	8	9	8	5	11	12	10	12	5
Drug-Drug Interactions	12	11	12	10	10	11	11	10	10	6	10	11	12	10	10
Emerging Resistance	12	8	9	4	10	0	0	9	10	6	2	8	9	10	0
Administration	10	10	8	8	8	6	6	10	10	7	9	10	10	9	4
Mechanism of Action	9	8	9	8	6	8	8	9	7	7	7	8	8	7	7
Food-Drug Interactions	7	7	5	4	4	2	2	4	4	3	4	7	5	4	2
Compatibility/Stability	7	4	4	0	3	0	0	4	4	0	3	4	5	4	0
Spectrum of Activity	7	5	6	4	5	3	3	5	5	6	4	5	5	7	4
Pregnancy/Lactation	7	5	7	6	5	5	5	6	6	5	5	7	6	6	5
Synergy	6	6	5	4	5	3	3	5	4	4	3	6	6	6	3
Drug-Herb Interactions	5	2	2	1	2	0	2	1	2	1	1	2	2	3	2
Cost	5	0	0	3	0	2	2	0	0	3	2	2	3	0	0
Pharmacokinetics	4	3	3	3	4	1	1	4	4	2	2	3	3	4	1

**Percent Answered (%)**		**78**	**77**	**65**	**65**	**46**	**47**	**73**	**71**	**56**	**65**	**81**	**81**	**82**	**41**

N = number of questions; AHFS = American Hospital Formulary Service Drug Information; CP = Clinical Pharmacology; DIO = DIOne; DM = DailyMed; EOF = Epocrates Online Free; EOP = Epocrates Online Premium; FC = Facts & Comparisons 4.0 Online; IDI = Internet Drug Index; JHAG = Johns Hopkins ABX Guide; LC = Lexi-Comp; LC-AHFS = Lexi-Comp with American Hospital Formulary Service; MDR = Medscape Drug Reference; MM = Micromedex, PPDC = PEPID PDC

### Completeness

Similar to the scores for scope, results for completeness were stratified into three distinct tiers including: Tier 1 (97-89%), Tier 2 (83-81%), and Tier 3 (74%), which were all significantly different from each other (p < 0.05). The top scoring databases for completeness were MM (97%), DM (96%), IDI (95%), MDR (95%), AHFS (94%), CP (94%), FC (94%), LC-AHFS (94%), and DIO (89%). Mid-ranking databases (Tier 2) were LC (83%), JHAG (82%), EOF (81%), and EOP (81%). PPDC (74%) scored in the lowest tier. Full results for completeness scores, including scores in each drug information category, are provided in Table 5.

**Table 5:** Completeness of databases

**CATEGORY**	**AHFS**	**CP**	**DIO**	**DM**	**EOF**	**EOP**	**FC**	**IDI**	**JHAG**	**LC**	**LC-AHFS**	**MDR**	**MM**	**PPDC**
Dosing	3.00	3.00	2.83	3.00	2.17	2.17	2.92	2.92	2.38	2.67	2.92	2.92	3.00	2.83
Indication	2.85	2.73	2.91	3.00	2.50	2.50	2.89	3.00	2.55	2.62	2.85	2.86	3.00	2.29
Adverse Reaction/Event	2.78	2.83	2.88	2.90	2.83	2.83	2.91	2.91	2.63	2.76	2.78	2.78	3.00	2.75
Contraindications	3.00	3.00	2.50	2.88	2.50	2.50	3.00	2.75	2.00	2.36	2.92	3.00	2.92	2.40
Drug-Drug Interactions	2.82	2.83	2.50	3.00	2.64	2.64	2.30	2.70	1.83	2.30	2.82	2.92	2.90	1.70
Emerging Resistance	2.75	2.67	3.00	3.00	N/A	N/A	3.00	3.00	2.67	2.00	2.88	2.89	3.00	N/A
Administration	3.00	3.00	2.63	3.00	2.67	2.67	2.90	2.90	2.67	2.56	3.00	2.90	3.00	2.50
Mechanism of Action	2.88	3.00	2.25	2.83	1.88	1.88	2.67	2.86	2.86	2.14	2.88	2.88	2.57	1.86
Food-Drug Interactions	2.43	2.80	2.50	2.75	2.00	2.00	3.00	3.00	2.33	2.75	2.43	2.60	3.00	N/A
Compatibility/Stability	3.00	2.50	N/A	2.67	N/A	N/A	2.75	2.75	N/A	2.33	3.00	3.00	3.00	N/A
Spectrum of Activity	3.00	2.67	3.00	3.00	2.33	2.33	3.00	3.00	3.00	3.00	3.00	3.00	3.00	3.00
Pregnancy/Lactation	2.40	2.86	2.33	2.20	2.00	2.00	2.67	2.50	2.40	2.80	2.57	2.00	2.50	1.80
Synergy	2.67	2.60	2.50	2.80	3.00	3.00	2.40	3.00	2.50	2.00	2.67	3.00	2.67	2.67
Drug-Herb Interactions	2.50	2.50	3.00	2.00	N/A	2.00	3.00	1.50	2.00	1.00	2.50	2.50	2.67	2.00
Cost	N/A	N/A	3.00	N/A	3.00	3.00	N/A	N/A	3.00	3.00	3.00	3.00	N/A	N/A
Pharmacokinetics	3.00	3.00	2.33	3.00	2.00	2.00	3.00	2.75	2.50	2.00	3.00	3.00	3.00	2.00

**(%)**	**94**	**94**	**89**	**96**	**81**	**81**	**94**	**95**	**82**	**83**	**94**	**95**	**97**	**74**

N = number of questions; AHFS = American Hospital Formulary Service Drug Information; CP = Clinical Pharmacology; DIO = DIOne; DM = DailyMed; EOF = Epocrates Online Free; EOP = Epocrates Online Premium; FC = Facts & Comparisons 4.0 Online; IDI = Internet Drug Index; JHAG = Johns Hopkins ABX Guide; LC = Lexi-Comp; LC-AHFS = Lexi-Comp with American Hospital Formulary Service; MDR = Medscape Drug Reference; MM = Micromedex, PPDC = PEPID PDC; N/A = not applicable

### Categorical analysis

**Figure 1:**
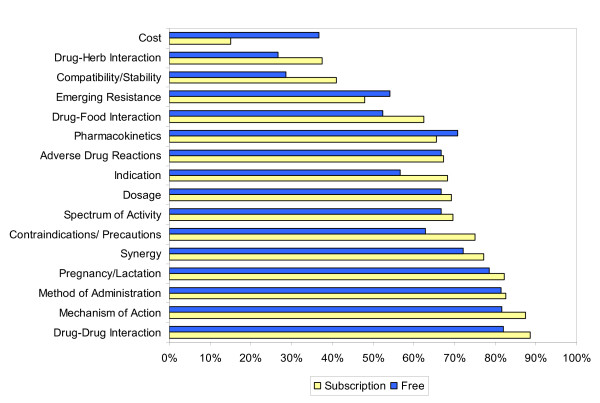
Scope comparison of drug information categories between subscription and free databases.

**Figure 2:**
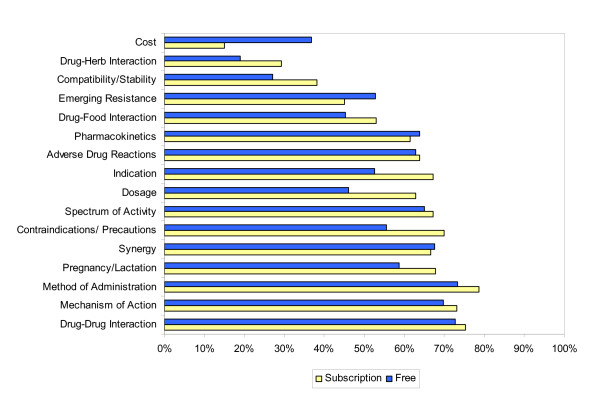
Completeness comparison of drug information categories between subscription and free databases.

When exclusively examining differences in the ability to answer questions (scope) in the ID categories, subscription databases performed better within both the ID-specific and non-ID specific categories than the free databases (p = 0.03). ID-specific categories included emerging resistance, spectrum of activity, and synergy. However, there was no difference between free and subscription online databases in scope between individual categories (e.g., dosing, administration, etc.). Comparisons of scope and completeness scores between free and subscription databases are shown in Figures 1 and 2, respectively.

### Sub-analysis of related databases

While no differences in scope were found between AHFS and LC-AHFS, both databases answered more questions than LC alone (p < 0.05). Similar findings were seen in regards to completeness, where AHFS and LC-AHFS were more complete in answering the questions than LC alone (p < 0.05), but no difference was found between AHFS and LC-AHFS. When comparing EOF and EOP databases, no differences in scope or completeness (p > 0.05) were seen.

### Errors

There were 37 erroneous answers found in this analysis yielding an overall error rate of 1.8%. Of the fourteen databases evaluated, three databases had no errors (MM, AHFS, and MDR), four had two errors (DM, FC, LC, and LC-AHFS), four had three errors (CP, DIO, IDI, and JHAG), and five errors were found in both versions of Epocrates. PPDC had seven wrong answers, which was found to be significantly higher than the other databases (p < 0.05). A summary of errors in each category is shown in Table 6, and a sample of erroneous answers that have the potential to impact clinical outcomes and patient safety has been provided in Table 7.

**Table 6:** Errors by database and errors per category

**CATEGORY**	**N**	**AHFS**	**CP**	**DIO**	**DM**	**EOF**	**EOP**	**FC**	**IDI**	**JHAG**	**LC**	**LC-AHFS**	**MDR**	**MM**	**PPDC**	**TOTAL ERRORS PER CATEGORY**
Dosing	15	-	-	-	-	-	-	-	-	2	-	-	-	-	2	**4**
Indication	15	-	2	1	1	-	-	1	-	1	1	1	-	-	3	**11**
Adverse Drug Reaction	13	-	-	-	-	2	2	-	2	-	-	-	-	-	-	**6**
Contraindications	13	-	-	1	1	1	1	1	1	-	1	1	-	-	1	**9**
Method of Administration	10	-	1	-	-	1	1	-	-	-	-	-	-	-	1	**4**
Drug-Food Interactions	7	-	-	1	-	1	1	-	-	-	-	-	-	-	-	**3**

**TOTAL ERRORS PER DATABASE**		**0**	**3**	**3**	**2**	**5**	**5**	**2**	**3**	**3**	**2**	**2**	**0**	**0**	**7**	

N = number of questions; AHFS = American Hospital Formulary Service Drug Information; CP = Clinical Pharmacology; DIO = DIOne; DM = DailyMed; EOF = Epocrates Online Free; EOP = Epocrates Online Premium; FC = Facts & Comparisons 4.0 Online; IDI = Internet Drug Index; JHAG = Johns Hopkins ABX Guide; LC = Lexi-Comp; LC-AHFS = Lexi-Comp with American Hospital Formulary Service; MDR = Medscape Drug Reference; MM = Micromedex, PPDC = PEPID PDC

**Table 7:** Sample of erroneous answers discovered in databases

**Category**	**Question**	**Correct answer used for assessment**	**Erroneous answer(s)**	**Database(s)**
Dosing	What is the recommended dosing regimen for fosamprenavir in a protease inhibitor-experienced HIV+ patient who wants once daily dosing?	Once daily dosing is not recommended for this type of patient	• 700 mg by mouth once daily plus ritonavir 100 mg twice daily for PI experienced patients	• PPDC

Adverse Drug Reaction	Can ethionamide cause impotence?	Yes, but it is a rare ADR.	• Listed as a common reaction	• JHAG

Method of Administration	How do you administer Synagis?	Remove flip top from Synagis vial and wipe rubber stopper with a disinfectant (e.g., 70% isopropyl alcohol). Insert needle into vial, and withdraw into syringe an appropriate volume of solution. Administer immediately after drawing dose into syringe. Synagis is supplied as single-dose vial and does not contain preservatives. Do not re-enter vial after withdrawal of drug; discard unused portion. Only administer one dose per vial.	• Provides information about scheduling multiple patients for multiple injections from same vial to minimize waste	• CP

Indication	Should amantadine be given as prophylaxis against influenza A?	It should not be used in the 2007–2008 influenza season due to resistance.	• Provides pediatric dosing information for this indication • Lists influenza A prophylaxis as an indication	• PPDC • DM • IDI

Drug-Food interaction	How should didanosine be taken in regards to food?	Didanosine should be taken on an empty stomach at least 30 minutes before or 2 hours after food. Do not take didanosine or didanosine EC with food.	• Provides contradictory information: States that didanosine can be taken with or without food in one sentence and the following statement states to take on an empty stomach 30 minutes before or 2 hours after food.	• EOF • EOP • DIO

CP = Clinical Pharmacology; DIO = DIOne; DM = DailyMed; EOF = Epocrates Online Free; EOP = Epocrates Online Premium; IDI = Internet Drug Index; JHAG = Johns Hopkins ABX Guide; PPDC = PEPID PDC

## Discussion

In a field as dynamic and evolving as ID, the use of CDSTs, such as drug information databases, can improve patient safety and clinical outcomes. However, a reference is only as good as the information it contains and this study revealed that improvements are necessary. Several general drug information databases were able to provide superior depth and breadth of information, while other references did not perform as well. MM (82%), MDR (81%), LC-AHFS (81%), AHFS (78%), and CP (77%) were all top performers, but even the database with the highest score for scope (MM) was unable to answer nearly one-fifth of the evaluative questions, and the database with the lowest scope score (PPDC) fell short by almost 60%. This deficit, in practical terms, shows that MM would be unable to provide an answer to one out of every five drug information queries, and PPDC would be unable to provide an answer to three out of every five. This disparity between the information needed by the healthcare professional and the information provided by the resources could have a far-reaching negative effect on both practical utility and clinical outcomes.

Not providing a sufficient scope of information is not as critical as providing accurate information. If an answer is not found, the healthcare provider can turn to another resource to locate the answer. But, if a wrong answer is given, the provider may not realize that the information is unreliable and use it to make a critical decision, potentially resulting in a negative outcome or even patient harm. The number of errors (1.8%) found amongst the databases was alarming. Out of the 14 references evaluated, only three (i.e. MM, AHFS, and MDR) returned no erroneous answers. Of the drug information categories in which errors were found, two (dosing and indication) were deemed so important to direct patient care and patient safety that they were the top two weighted categories in this study, both with 15 questions (10.2%) each. While some of the errors were blatantly wrong, others were instances where the database gave information that was not in line with that provided by the manufacturer but may be acceptable in current clinical practices. CDSTs have been proven to be useful in making timely and accurate patient care decisions at all stages of the decision-making process, but this study has shown that CDTSs cannot be considered reliable all of the time. Healthcare providers, in all aspects of practices, are expected to be accurate 100% of the time in order to ensure patient safety. This goal cannot be achieved if the tools that are used in the course of practice are not held to that same high standard.

## Limitations

Our study had several potential limitations. The evaluative questions used were intended to be a subset of all possible drug information questions. While we were careful to represent as many types of drug information questions as possible, inclusion of all clinical aspects was not feasible. Performing the same evaluation with a different set of questions could produce different results; however, because of the broad range of scenarios represented in the original question list, the results would most likely yield little or no differences. Also, our study captured the data available from the databases at a set point in time, but the databases are tools that are updated with varying frequency. It is possible that changes have been made to the information contained in the databases since the time of this evaluation. And finally, some publishers offer additional components with their drug information databases (e.g., dose calculators). If these value-added features required an additional purchase, they were not used for this evaluation.

## Conclusion

Drug information databases used in ID practices as CDSTs can be valuable resources for the healthcare provider. MM, MDR, LC-AHFS, AHFS, and CP were shown to be superior in their scope and completeness of information, and MM, AHFS, and MDR provided no erroneous answers. There is room for improvement in all databases evaluated in this study.

## Competing interests

HHP, AZ, JJ, and MP declare that they have no competing interests. KAC has received grant support from Elsevier Science/Gold Standard, Inc. which produces Clinical Pharmacology.

## Authors' contributions

HHP conceived the project, developed the study design, wrote the question list, performed data collection, and wrote and critically edited the manuscript. AZ contributed to the study design and question list, conducted the statistical analysis, and wrote and critically edited the manuscript. KAC contributed to the study design and wrote and critically edited the manuscript. JJ wrote the question list, assisted with data collection, and critically edited the manuscript. MP assisted in question development and critically edited the manuscript. All authors read and approved the final manuscript.

## Pre-publication history

The pre-publication history for this paper can be accessed here:


